# Increased Crystallization of CuTCNQ in Water/DMSO Bisolvent for Enhanced Redox Catalysis

**DOI:** 10.3390/nano11040954

**Published:** 2021-04-08

**Authors:** Zakir Hussain, Ayman Nafady, Samuel R. Anderson, Abdullah M. Al-Enizi, Asma A. Alothman, Rajesh Ramanathan, Vipul Bansal

**Affiliations:** 1 Ian Potter NanoBioSensing Facility, NanoBiotechnology Research Laboratory (NBRL), School of Science, RMIT University, P.O. Box 2476, Melbourne, VIC 3000, Australia; s3472654@student.rmit.edu.au (Z.H.); s3239826@student.rmit.edu.au (S.R.A.); 2 Chemistry Department, College of Science, King Saud University, Riyadh 11451, Saudi Arabia; amenizi@ksu.edu.sa (A.M.A.-E.); aaalothman@ksu.edu.sa (A.A.A.)

**Keywords:** metal–organic semiconductor, CuTCNQ, charge-transfer complex, co-solvent, redox catalysis

## Abstract

Controlling the kinetics of CuTCNQ (TCNQ = 7,7,8,8-tetracyanoquinodimethane) crystallization has been a major challenge, as CuTCNQ crystallizing on Cu foil during synthesis in conventional solvents such as acetonitrile simultaneously dissolves into the reaction medium. In this work, we address this challenge by using water as a universal co-solvent to control the kinetics of crystallization and growth of phase I CuTCNQ. Water increases the dielectric constant of the reaction medium, shifting the equilibrium toward CuTCNQ crystallization while concomitantly decreasing the dissolution of CuTCNQ. This allows more CuTCNQ to be controllably crystallized on the surface of the Cu foil. Different sizes of CuTCNQ crystals formed on Cu foil under different water/DMSO admixtures influence the solvophilicity of these materials. This has important implications in their catalytic performance, as water-induced changes in the surface properties of these materials can make them highly hydrophilic, which allows the CuTCNQ to act as an efficient catalyst as it brings the aqueous reactants in close vicinity of the catalyst. Evidently, the CuTCNQ synthesized in 30% (*v*/*v*) water/DMSO showed superior catalytic activity for ferricyanide reduction with 95% completion achieved within a few minutes in contrast to CuTCNQ synthesized in DMSO that took over 92 min.

## 1. Introduction

The rich electrical, chemical, magnetic, and optoelectronic properties of charge-transfer complexes based on 7,7,8,8-tetracyanoquinodimethane (TCNQ) has seen substantial interest in using these materials for a range of applications (Figure S1, Supplementary Materials show the chemical structure of TCNQ) [1,2,3,4,5,6,7,8]. The pioneering work led by Dunbar, Miller, Robson, and Bond have seen the development of several strategies including physical [9,10], photochemical [11,12,13], vapor deposition [9,14,15], electrochemical [5,13,16,17,18,19,20,21,22], and wet-chemical synthesis methods [19,23,24,25,26,27,28,29,30,31,32,33,34,35,36] for the fabrication of TCNQ-based charge transfer complexes. The resultant materials have been used as sensors [14,15,37,38], photocatalysts [19,23,24,25,26,29,30,31,32,33,35,36], data storage systems [39,40], super-hydrophobic surfaces [18,41,42], antibacterial materials [27], high-capacity cathodes for K^+^ and Na^+^ ion battery [43,44,45], and electrocatalysts for hydrogen and oxygen evolution reactions [46,47,48]. Owing to the wider potential applicability of these materials, there has been a recent emergence of interest in improving their fabrication strategies.

Much of the work in the area has focused on MTCNQ (M = Cu, Ag) where a simple wet-chemical approach has been used for its synthesis (Figure S2, Supplementary Materials) [13,30]. Specifically, the fabrication of phase I CuTCNQ using this approach involves exposing a piece of Cu foil (Cu^0^) to acetonitrile (MeCN) solution of neutral TCNQ^0^ at or close to ambient temperature. A spontaneous redox reaction results in the oxidation of metallic Cu^0^ to Cu^+^ ion and reduction of TCNQ^0^ to TCNQ^1−^ ion, leading to the crystallization of CuTCNQ on the surface of the metal foil [13,30,32,33,35]. However, this well-established route has a limitation—during the fabrication of CuTCNQ in MeCN, two parallel processes occur, viz. crystallization of CuTCNQ on Cu foil and corrosion/dissolution of CuTCNQ in MeCN from the surface of the Cu foil to produce Cu^+^ and TCNQ^−^ ions. An equilibrium between the two (crystallization/corrosion) processes limits the fabrication of larger CuTCNQ structures, which is a key aspect for several applications [32]. A potential strategy to shift the equilibrium of the reaction would be to increase the temperature of the reaction during synthesis. However, in the case of CuTCNQ, increasing the reaction temperature and exposing CuTCNQ to higher temperatures for a prolonged time results in a physicochemical change in the material where the conductive phase I CuTCNQ gets converted to less conductive phase II CuTCNQ [7,35]. This would mean that the resulting CuTCNQ would not be equally suitable for electronics or catalysis applications. For instance, phase I CuTCNQ has been shown to be a good catalyst that is useful for the photocatalytic degradation of organic dyes [23,24], reduction of ferricyanide [30,32,33,35], and reduction of hexavalent chromium [25]. In contrast, some of these reactions were significantly slow when less conductive phase II CuTCNQ was employed as a catalyst [35].

Having realized this limitation, our team has focused on developing new strategies to control the crystallization/corrosion process. We previously noted that a multi-step approach where Cu foil is repeatedly exposed to fresh TCNQ^0^ solution during the reaction results in an equilibrium shift toward enhanced CuTCNQ crystallization. This is because, after the addition of fresh TCNQ^0^, the reaction would contain more of the TCNQ^0^ species than TCNQ^−^ species [34]. This approach resulted in structures of over 50 µm, which was previously limited to 10–15 µm through conventional wet-chemical synthesis [19,23,24,25,26,27,28,29,30,31,32,33,34,35,36]. Furthermore, we proposed the use of a bisolvent mixture where a small fraction of water was mixed with TCNQ^0^ dissolved in MeCN. This resulted in an equilibrium shift and promoted the crystallization of CuTCNQ, as this material is minimally soluble in water [32]. To this end, it is important to note that the precursor TCNQ^0^ is insoluble in water; thus, synthesis is not possible in pristine water as a solvent. As such, this approach showed CuTCNQ crystals of ≈100 µm in length where the large structures significantly improved the catalytic efficiency of redox reactions [32]. More recently, we reported that the choice of solvent in which the synthesis occurs also had a large influence on the crystallization of CuTCNQ. Interestingly, this latest work revealed that the dielectric constant of the solvent not only plays an important role in the CuTCNQ crystallization process, but it also suggested that the choice of solvent influences the charge transfer properties of CuTCNQ when employed as catalysts. For instance, CuTCNQ fabricated in dimethyl sulfoxide (DMSO) showed better catalytic performance for redox reactions than CuTCNQ grown in MeCN or dimethylformamide (DMF) [35]. This suggests that the choice of solvent and shifting the equilibrium toward CuTCNQ crystallization are both important considerations to fabricate effective catalysts.

In the current work, we combine the learnings from our previous reports, such that we employ DMSO as the solvent phase to produce CuTCNQ with inherently enhanced catalytic properties, while employing water as a co-solvent to shift the reaction equilibrium toward promoting the CuTCNQ crystallization on the surface of the Cu foil. The increase in the crystallization process results in the length and width of the CuTCNQ crystals to increase significantly. The increase in the CuTCNQ crystallization further results in improving the catalytic rate of redox catalysis reaction between ferricyanide and thiosulfate where the hydrophobicity of surface was one of the factors responsible for the improved rate. This study opens up new avenues for controlling the crystal growth of MTCNQ-based charge-transfer complexes by establishing the universality of water as a co-solvent.

## 2. Materials and Methods

### 2.1. Materials and Reagents

Copper foil (99% pure), DMSO, sodium thiosulphate, and potassium (III) ferricyanide were purchased from Sigma Aldrich, Australia. 7,7,8,8-tetracyanoquinodimethane (TCNQ) was purchased from Chem Supply. Deionized water (18.2 MΩ cm) was dispensed from Millipore Milli-Q Ultrapure Water system fitted with Organex-Q Cartridge filters. All chemicals were used as received.

### 2.2. Synthesis of Phase I CuTCNQ

A 1 × 1 × 0.05 cm^3^ Cu foil was treated with dilute nitric acid (0.1 M) for approximately 20 s to remove surface oxide impurities. The foil was washed with DI water, dried under nitrogen gas, and used further. The Cu foil was exposed to DMSO solution containing 1, 2.5, and 5 mM dissolved TCNQ^0^. The reaction between Cu foil and TCNQ was carried out for 1 h at 25 °C. The reaction mechanism of CuTCNQ formation is very well known. In brief, the Cu^0^ from Cu foil reacts with neutral TCNQ in appropriate solvent where the reaction proceeds via a redox reaction given by

(1) $${\mathrm{Cu}}^{0}+{\;\mathrm{TCNQ}}^{0}\;⇌{\;\mathrm{Cu}}^{+}{\mathrm{TCNQ}}^{-}\;⇌\mathrm{CuTCNQ}.\;$$

Parallel reactions under similar conditions were performed in bisolvent mixtures containing 5, 15, and 30% *v/v* water with respect to DMSO. After the reaction, the Cu foil containing CuTCNQ structures was removed, washed with DMSO and DI water, dried under nitrogen gas, and stored in a vacuum desiccator for further use.

### 2.3. Material Characterization

The authors thank the RMIT Microscopy and Microanalysis Facility (RMMF) for their technical assistance and providing access to materials characterization facilities. SEM images of the samples were obtained using FEI Verios 460L FE-SEM instrument operated at an accelerating voltage of 10 kV; energy dispersive X-ray (EDX) analysis was performed on an FEI Verios 460 L FE-SEM coupled with EDX Oxford X-Max Silicon Drift Detector; Raman spectroscopy was carried out using a Perkin-Elmer Raman Station 200F at an excitation wavelength of 785 nm and 100 μm spot size. FTIR spectroscopy was performed using a Perkin–Elmer D400 spectrophotometer in attenuated total reflectance mode with a resolution of 4 cm^−1^. The UV-Vis spectroscopic studies were carried out with Cary 50 Bio spectrophotometer. Water contact angles were performed using an OCA20 contact-angle system at ambient temperature.

### 2.4. Dissolution Studies in Different Solvents

The dissolution of CuTCNQ was studied by placing prefabricated CuTCNQ in DMSO, MeCN, and water for 1 h at 25 °C. After 1 h, the Cu foil containing the CuTCNQ was removed, and the solution was analyzed using UV-visible spectroscopy.

### 2.5. Catalysis Experiments Using CuTCNQ as Catalyst

The catalytic property of CuTCNQ was tested using 0.1 M sodium thiosulphate and 1 mM potassium ferricyanide in a 30 mL reaction volume at 25 ± 2 °C under constant stirring conditions. For this, ≈0.7 mg of CuTCNQ was scrapped from the surface of each CuTCNQ foil and added into the reaction vessel. The reaction vessel was covered with aluminium foil to avoid the potential influence of ambient light in photoexciting the material. The catalytic conversion of ferricyanide into ferrocyanide was monitored as a function of time using UV-Vis absorbance spectroscopy.

## 3. Results and Discussion

**Figure 1:**
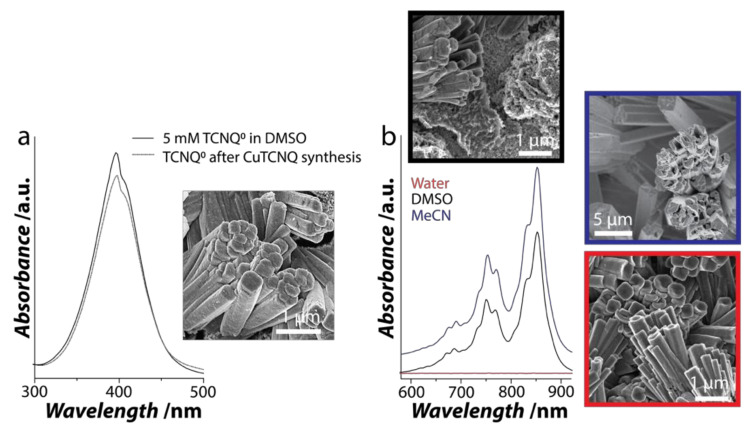
Crystallization and dissolution of CuTCNQ. (**a**) UV-Vis spectra of TCNQ^0^ solution in DMSO before and after CuTCNQ synthesis and the SEM image of CuTCNQ formed on the Cu foil via crystallization in DMSO (**b**) UV-Vis absorbance spectra and the SEM images obtained after exposure of CuTCNQ to MeCN, DMSO and water for one hour at room temperature. TCNQ = 7,7,8,8-tetracyanoquinodimethane.

While MeCN is most commonly employed as the reaction medium for the synthesis of CuTCNQ, our previous work has shown the potential of DMSO as a preferred reaction solvent for CuTCNQ crystallization due to the superior catalytic performance of CuTCNQ synthesized in DMSO over that in MeCN [35]. To understand the influence of water/DMSO bisolvent on CuTCNQ crystallization, the first step was to understand the two parallel processes that occur during synthesis viz. (i) the crystallization of CuTCNQ in DMSO and (ii) the dissolution of CuTCNQ in different solvents. For this, the synthesis of CuTCNQ was carried out using a wet chemical route where a pre-cleaned Cu foil was immersed for 1 h in DMSO containing 5 mM of dissolved TCNQ^0^. A spontaneous one-electron reaction occurs between Cu^0^_(foil)_ and TCNQ^0^_(DMSO)_. This resulted in the crystallization of CuTCNQ on Cu foil. The UV-visible spectra obtained from DMSO solutions before and after TCNQ synthesis provided a good indication of the amount of TCNQ^0^ consumed during the synthesis. In particular, the UV-visible absorbance features between the 300 and 500 nm range are characteristic of TCNQ^0^ (Figure 1a) [27,28,32,33,34,35]. The UV-visible spectrum from pristine TCNQ dissolved in DMSO showed a distinct peak at ca. 398 nm, which can be attributed to TCNQ^0^. Following the synthesis of CuTCNQ, the UV-visible spectrum showed a decrease in the peak intensity at ca. 398 nm (Figure 1a dotted line). This suggests that TCNQ^0^ was consumed during the crystallization of CuTCNQ (≈10% TCNQ consumed during synthesis). The formation of CuTCNQ was also evident from the change in the color of the solution from yellow to green, which occurs due to the co-dissolution of freshly formed CuTCNQ into Cu^+^ and TCNQ^−1^ species (Figure S3, Supplementary Materials).

The SEM image of the resulting CuTCNQ structures showed typical rod-like morphology with 5–10 μm in length and 100–300 nm in diameter (inset in Figure 1a). Consistent with our previous work, we observed that the packing density of the CuTCNQ microrods was higher than that observed when CuTCNQ is grown in MeCN. Next, we assessed the dissolution of prefabricated CuTCNQ in DMSO, MeCN, and water by immersing the Cu foil containing CuTCNQ synthesized in DMSO into different solvents for 1 h. The UV-visible spectra obtained from the solutions after removing the Cu foil showed several peaks in the 600–900 nm region, which are characteristic features of TCNQ^−^ (Figure 1b) [27,28,32,33,34,35]. Based on the peak intensities, it is evident that the dissolution of CuTCNQ to Cu^+^ and TCNQ^−^ in DMSO was less than that in MeCN. In contrast, no feature corresponding to TCNQ^−^ was observed in the sample exposed to water, suggesting that CuTCNQ has poor solubility in water. The SEM image obtained from the CuTCNQ sample exposed to DMSO had a significant effect on the morphology where the dissolution results in smaller irregular structures on the surface of the Cu foil. This observation is starkly different from the CuTCNQ exposed to MeCN where the dissolution of the CuTCNQ leads to hollow rods. Consistent with the UV-Vis spectrum, the SEM image obtained from the CuTCNQ sample exposed to water showed no or minimal change in the morphology.

Our previous work has shown that to increase the size of the CuTCNQ structures, one of the key considerations is to shift the reaction equilibrium toward the crystallization of CuTCNQ and away from the co-dissolution in the synthesis media. This was achieved by the addition of water, as water reduces the CuTCNQ dissolution [32]. Our subsequent work further suggested that the dielectric constant of the synthesis medium is also a key aspect to shift this equilibrium in favor of crystallization [35]. Considering that the dielectric constant of water is ≈80, while that of DMSO and MeCN is ≈46.7 and 37.5, respectively, it is possible that in the former study where a bisolvent approach was used, mixing MeCN with water resulted in a shift in the dielectric constant of the system such that the reaction favored CuTCNQ crystallization. To assess if this phenomenon is universally applicable, we fabricated CuTCNQ on Cu foil using TCNQ^0^ dissolved in DMSO containing 0%, 5%, 15%, and 30% *v*/*v* water. To understand the process of crystallization, three independent concentrations of TCNQ^0^ viz. 1, 2.5, and 5 mM were used. The fabrication was performed at room temperature for one hour, while the dimension of the Cu foil was kept constant in all cases. A key aspect in the water/DMSO bisolvent strategy was to ensure that the reactant TCNQ^0^ does not precipitate into the aqueous phase. DMSO and water are highly miscible where water acts as a hydrogen bond donor while DMSO is hydrogen bond accepter [49,50]. As the concentration of water was increased in the solution containing TCNQ^0^ dissolved in DMSO, the hydrogen bonding between water and DMSO molecules increases. This results in the precipitation of TCNQ^0^ at high water concentration. Therefore, the highest water concentration employed in our study was 30%, where no precipitation of TCNQ was observed in the solution.

**Figure 2:**
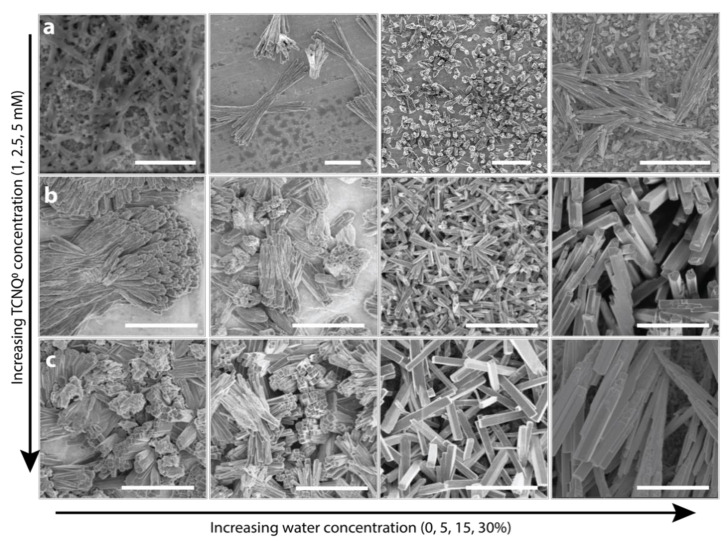
Material characterization of CuTCNQ. SEM images of CuTCNQ grown on Cu foil using (**a**) 1 mM, (**b**) 2.5 mM, and (**c**) 5 mM TCMNQ in DMSO with increasing concentration of water (0, 5, 15, 30% *v*/*v*). The scale bar is 5 µm except for (**a**) 1 mM 0% H_2_O which is 500 nm.

Figure 2 illustrates the SEM images of CuTCNQ synthesized in 1 mM (panel a), 2.5 mM (panel b), and 5 mM (panel c) TCNQ solution containing increasing concentration of water viz. 0%, 5%, 15%, and 30% *v*/*v*, respectively (additional low magnification images are shown in Figure S4, Supplementary Materials). During the crystallization of CuTCNQ in 1 mM TCNQ dissolved in DMSO (no water), the surface shows interconnected channels of CuTCNQ nanorods (Figure 2a). When a low concentration of water is introduced (5% *v*/*v*), discrete clusters of CuTCNQ with rod-like morphology were observed. These structures typically appear as aggregated nanorods where the length of each cluster was around 8–10 µm. When the concentration of water was further increased to 15% *v*/*v*, we observed a decrease in the size of the crystals to 2–4 µm in length, while there was a concomitant increase in the coverage of CuTCNQ clusters on the surface of the Cu foil. When the concentration of water was increased to 30% *v*/*v*, the coverage of CuTCNQ was observed to be across the Cu foil, while the crystals were consisted of 5–15 µm in length. Based on the SEM study, it can be inferred that with increasing concentration of water in the water/DMSO bisolvent, the crystallization of CuTCNQ on the surface of the Cu foil is enhanced. When the concentration of TCNQ^0^ dissolved in DMSO was increased to 2.5 mM, the surface coverage of CuTCNQ on the Cu foil increased (Figure 2b). This is expected as more TCNQ^0^ is now available to react with the Cu foil. The CuTCNQ fabricated in pure DMSO (0% water) showed clusters of CuTCNQ nanorods with an approximate length of 10–12 µm. When the concentration of water in the bisolvent mixture is increased to 5%, not only did the packing density of CuTCNQ clusters increase, the length of the CuTCNQ rods in the clusters decreased to ca. 6–8 µm. Further increasing the water concentration to 15% resulted in discrete CuTCNQ rods with an approximate length of 2–4 µm. At 30% *v*/*v* water concentration, the length of the CuTCNQ rods increased to 10–20 µm. When the concentration of TCNQ^0^ dissolved in DMSO was further increased to 5 mM, the trend was similar to that observed in 2.5 mM TCNQ^0^. However, the length of the rods and packing density increased. For instance, the rods obtained at the highest water concentration of 30% *v*/*v* were considerably longer with a length of ca. 30–40 µm and width of ca. 0.8–1 µm. Overall, the SEM study revealed some interesting observations: (i) the size of the rods initially decreases when a small amount of water is introduced in DMSO irrespective of the TCNQ^0^ concentration—this is most likely because as water shifts the equilibrium toward CuTCNQ crystallization, it results in additional nucleation sites on the surface of Cu foil, leading to decrease in the crystal size; (ii) there is a morphological change at 15% *v*/*v* water concentration in all three TCNQ^0^ concentrations where clusters of CuTCNQ change to CuTCNQ rods—this is possibly due to optimal growth conditions; and (iii) in addition to the increase in length, the addition of water also increased the width of the CuTCNQ—Figure S5 in the Supplementary Materials shows the width of CuTCNQ rods in each case (measured from 30–40 individual CuTCNQ rods)—this suggests a shift in the equilibrium in favor of CuTCNQ crystallization.

**Figure 3:**
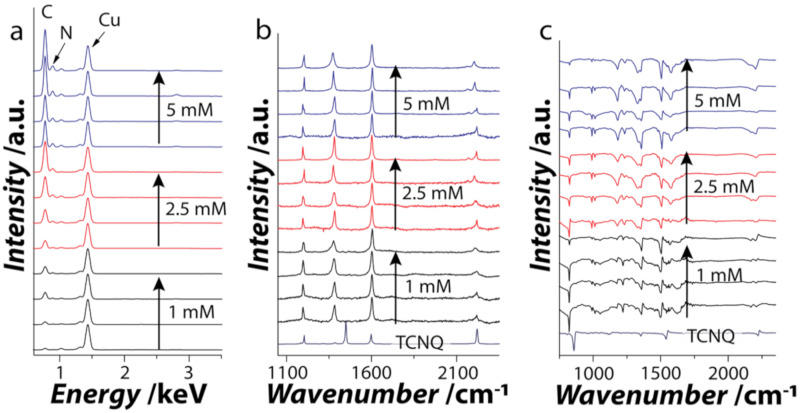
Spectroscopic characterization of CuTCNQ. (**a**) Energy dispersive X-ray (EDX), (**b**) Raman and (**c**) Fourier-transform infrared (FTIR) spectra of CuTCNQ grown on Cu foil using 1 mM, 2.5 mM, and 5 mM TCNQ in DMSO with increasing concentration of water (0, 5, 15, 30%).

Energy dispersive X-ray (EDX) spectra obtained from the CuTCNQ crystals in all cases show characteristic energy lines corresponding to C Kα, N Kα, and Cu Lα (0.27, 0.39, and 0.94 eV, respectively) (Figure 3a). The C Kα and N Kα energy lines can be assigned to TCNQ^−^ in CuTCNQ [27,28,32,33,34,35]. The absence of an O Kα energy line suggests that there is no or negligible oxidation of the Cu during the reaction. It is also clear that the intensity of the energy line corresponding to C Kα increases with the increase in the water concentration for each TCNQ^0^ concentration (relative to normalized Cu signal), further suggesting that the addition of water indeed shifts the equilibrium toward CuTCNQ crystallization. The EDX elemental mapping further confirmed the uniform distribution of CuTCNQ on the surface of the Cu foil (Figure S6, Supplementary Materials). CuTCNQ crystals was further characterized using Raman and Fourier-transform infrared (FTIR) spectroscopies. Raman spectroscopy can readily identify the difference between neutral TCNQ^0^ and TCNQ^−1^ anion, confirming the formation of the charge transfer CuTCNQ complex [27,28,32,33,34,35], while FTIR can provide information about the phase of CuTCNQ obtained [27,28,32,33,34,35]. The background corrected Raman spectrum [51] of pristine TCNQ shows characteristic peaks corresponding to the C-CN wing stretch at 1450 cm^−1^ and C≡N wing stretch at 2225 cm^−1^ (Figure 3b). The formation of CuTCNQ results in a shift in these peaks as the TCNQ^0^ interacts with Cu through its nitrile group [27,28,32,33,34,35]. The C-CN wing stretch peak shows a red shit from 1450 cm^−1^ to 1380 cm^−1^, while a shift in the C≡N stretch from 2225 cm^−1^ to 2200 cm^−1^ indicates the formation of CuTCNQ. No peaks corresponding to TCNQ^0^ were observed in the samples containing CuTCNQ. FTIR spectra showed characteristic peaks for C-H bending vibrations at ca. 824 cm^−1^, C=C wing stretching at 1505 cm^−1^, and C≡N stretching vibrations at 2200 cm^−1^ with a shoulder peak around 2172 cm^−1^ (Figure 3c). These FTIR features are characteristic of TCNQ^−1^ and not TCNQ^0^ or TCNQ^−2^ [27,28,32,33,34,35], validating the formation of CuTCNQ in water/DMSO bisolvent. Further, the phase of CuTCNQ was identified to be phase I as the C≡N stretching vibrations were observed at 2200 cm^−1^ instead of 2215 cm^−1^, which is otherwise a characteristic of phase II CuTCNQ [35].

**Figure 4:**
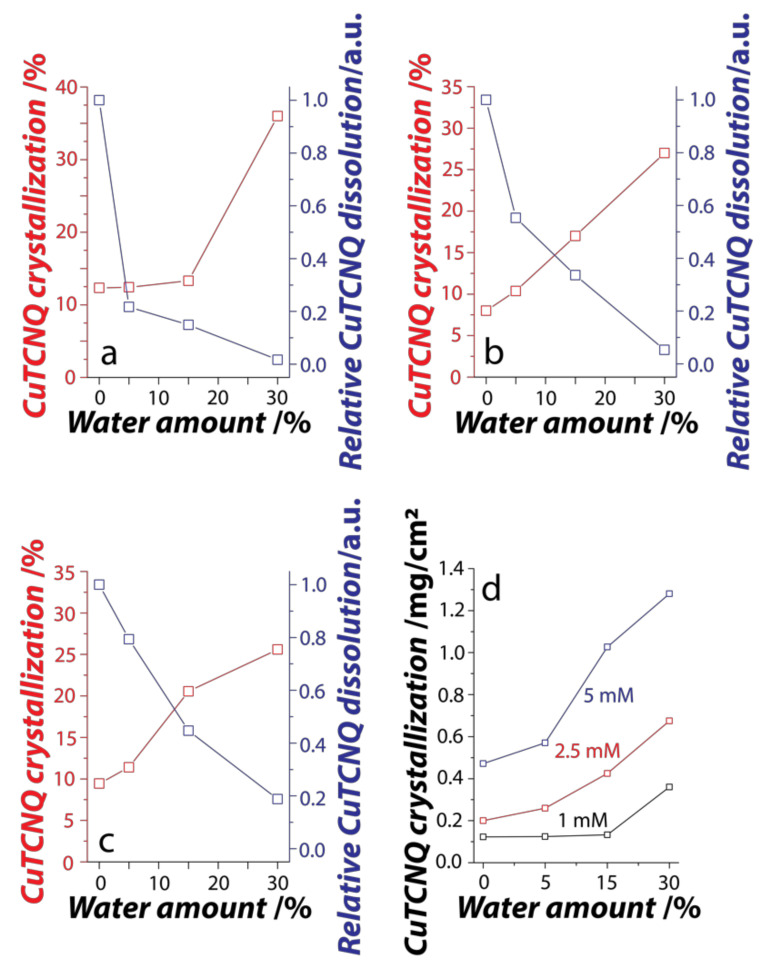
Crystallization and simultaneous co-dissolution of CuTCNQ in Water–DMSO bisolvent. Influence of water on CuTCNQ crystallization and corrosion at (**a**) 1 mM (**b**) 2.5 mM and (**c**) 5 mM TCNQ concentrations; (**d**) CuTCNQ crystallization on the surface of the Cu foil. % CuTCNQ crystallization in (**a**–**c**) refers to the proportion of TCNQ^0^ reactant used during the crystallization process, while the relative CuTCNQ dissolution in (**a**–**c**) compares dissolution in different amounts of water with respect to that in the absence of water, where maximum dissolution is expected.

To understand the influence of water on the overall crystallization process, we employed UV-Vis spectroscopy to assess the consumption of TCNQ^0^ from the reaction solvent during its crystallization into CuTCNQ on the Cu foil and the concomitant dissolution of CuTCNQ in the reaction solvent. The crystallization process can be studied by monitoring the reaction solution before and after the synthesis of CuTCNQ where the reduction in TCNQ^0^ signatures will correspond to TCNQ^0^ consumption, while an increase in TCNQ^–^ signature will reveal the dissolution of CuTCNQ (Figure S7, Supplementary Materials). A plot of the CuTCNQ crystallization and its relative dissolution is shown in Figure 4a–c. In all three concentrations of TCNQ^0^, we observe an increase in the crystallization of CuTCNQ as the concentration of water is increased (calculated using the consumption of TCNQ^0^—additional information in Experimental details). This observation is consistent with the SEM images (Figure 2) that show an increased coverage of CuTCNQ growth on the surface of the Cu foil as the concentration of water is increased during the reaction. Concurrently, we observe a decrease in the dissolution of CuTCNQ as the concentration of water is increased. It is interesting to observe that the dissolution reduced much more rapidly at lower concentrations of TCNQ^0^ where even 5% water in 1 mM TCNQ^0^ resulted in approximately 80% reduction in CuTCNQ dissolution. In contrast, a 20% reduction in dissolution was observed when the reaction was carried out in 5 mM TCNQ^0^ dissolved in DMSO containing 5% water. This is most possibly due to the difference in the total amount of CuTCNQ crystallized on the surface of the Cu foil in these reaction conditions. This becomes further evident from the calculation of the weight of CuTCNQ crystallized per cm^2^ of the Cu foil during different reaction conditions (Figure 4d). When the reaction proceeds at 1 mM (0.2 mg/mL TCNQ), a sharp increase in the amount of CuTCNQ crystallized is only observed at 30% water, while only a marginal increase is observed at 5–15% water concentrations. This increase equates to a 270% increase in the crystallization. In contrast, when the reaction proceeds at 2.5 mM (0.5 mg/mL TCNQ) and 5 mM (1 mg/mL TCNQ), the amount crystallized in comparison to no water was 225% and 185%, respectively. Although we see more TCNQ crystallized when the initial concentration of TCNQ^0^ in the reaction is more, the percent increase in crystallization is the highest at lower TCNQ^0^ concentrations with 30% water. This is possibly one of the reasons for the sharp reduction in the dissolution when the reaction proceeds at 1 mM TCNQ^0^ concentration as an equilibrium condition between crystallization and dissolution is achieved much more rapidly.

Based on our previous work, we know that the solvent in which CuTCNQ is synthesized plays a major role in its catalytic ability [35]. For instance, CuTCNQ synthesized in DMSO was found to be catalytically more active than CuTCNQ synthesized in MeCN [35]. For a catalyst to be effective in water-based reactions, the surface of the catalyst needs to be hydrophilic to ensure that the reactants come in close contact with the surface of the catalyst. To understand how the morphology of CuTCNQ crystallized on the surface of the Cu foil influences the solvophilic properties, we measured the water contact angle using the sessile drop method (Figure S8, Supplementary Materials). We noticed that for CuTCNQ synthesized using 1 mM TCNQ^0^, the hydrophobicity of the surface increased, as evident from an increase in the contact angle from 91.8° to 110.9° with increasing concentration of water. A similar trend was observed for CuTCNQ synthesized using 2.5 mM TCNQ^0^, where the increased hydrophobicity is evident from the change of contact angle from 92.4° at 0% *v*/*v* water to 102.1° at 15% *v*/*v* water. Interestingly, at 30% *v*/*v* water, the CuTCNQ surface showed a decrease in anti-wetting behavior with a contact angle of 98°. When CuTCNQ was synthesized using 5 mM TCNQ^0^, we observed an increase in the contact angle from 98.6° to 106.9° when the concentration of water was increased to 5% *v*/*v*. However, the contact angle decreased drastically to 66.5° and 14° for 15% and 30 % *v*/*v* water concentration, respectively, making these surfaces superhydrophilic. It is known that the creation of hydrophobic surfaces requires densely packed micro and nanoscale topologies and the presence of low surface energy [18,52,53]. The surface roughness can trap air, causing an increase in the water contact angle, while the low surface energy decreases the tendency of water to bond with the surface [18,52,53]. Based on the low magnification SEM images shown in Figure S6, we can see that in samples where there was a drop in the contact angle, irregular surface structures were observed due to the significant difference in the size of the CuTCNQ rods. Although this creates surface roughness, the density of packing is lower in these cases.

**Figure 5:**
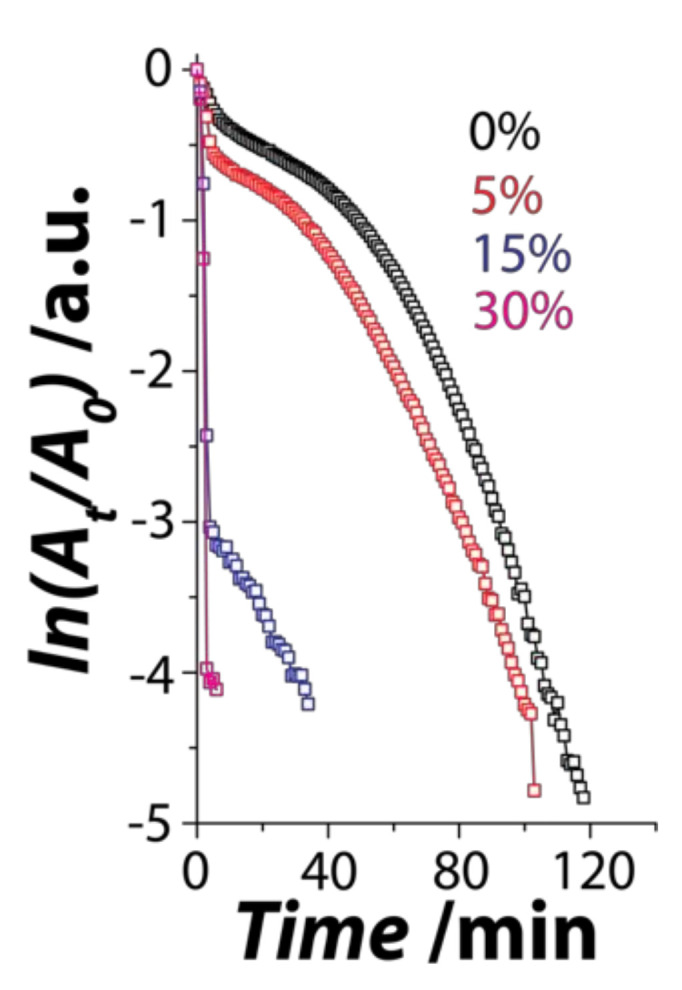
Redox catalysis using CuTCNQ catalyst. Time-dependent catalytic activity of CuTCNQ catalyst fabricated in 0%, 5%, 15%, and 30% *v*/*v* water, plotted as *ln*(*A_t_*/*A*_0_) versus the reaction time, where *A_t_* and *A*_0_ are absorbance values of ferricyanide at time = *t* and at 0, respectively.

Considering that the CuTCNQ samples fabricated using 5 mM TCNQ^0^ showed good hydrophilicity, we assessed these samples as a catalyst for a pseudo first-order redox reaction in which ferricyanide is reduced to ferrocyanide in the presence of excess thiosulfate ions. This reaction can be easily monitored using UV-Vis absorbance spectroscopy by measuring the change in the absorbance intensity of ferricyanide at 420 nm [30,32,33,35,36]. For all the catalytic reactions, the amount of catalyst was kept constant at ca. 0.7 mg. All samples showed the ability to reduce ferricyanide to ferrocyanide in the presence of excess thiosulfate (Figure 5 and Figure S9, Supplementary Materials). However, the catalytic efficiency in each case was different. A plot of the *ln*(A_t_/A_0_) versus time (where *A_t_* is the intensity of the absorbance at time t and *A_0_* is the intensity at time zero) was used to understand the reaction kinetics of the catalytic reactions. It is clear that introduction of water in DMSO during synthesis significantly influences the catalytic performance of the CuTCNQ catalyst. For instance, CuTCNQ synthesized in DMSO in the absence of water took ≈92 min to achieve a 95% reaction completion. When the CuTCNQ was synthesized in 5% *v*/*v* water, the catalytic activity improves, taking 81 min for 95% completion. When the CuTCNQ catalyst was synthesized at higher concentrations of water viz. 15% and 30% *v*/*v*, the catalytic performance improved significantly with only 4 and 2.5 min, respectively, which was taken to achieve 95% reduction. Comparison of the pseudo first-order apparent reaction rates (Table 1) revealed that CuTCNQ synthesized in 30% *v*/*v* water had a reaction rate of 1.89 min^−1^. This increase in the catalytic performance can be attributed to the synthesis condition as well as the highly hydrophilic nature of the material, which allows the ferricyanide and thiosulfate ions to be in close vicinity of the catalyst. The chemical stability of the CuTCNQ catalyst after the reaction was also assessed using FTIR spectroscopy, which showed no change in the spectra after the catalyst was used for the redox catalysis reaction (Figure S10, Supplementary Materials). This suggests that the catalyst remains highly stable.

## 4. Conclusions

In summary, this work establishes the utility of water as a simple co-solvent with DMSO to improve the spontaneous crystallization of CuTCNQ on the surface of Cu foil. The addition of water to DMSO increases the dielectric constant of the reaction medium which in turn results in reducing the rate of CuTCNQ co-dissolution during its synthesis, while concomitantly increasing the crystallization of CuTCNQ. Importantly, the addition of water to DMSO during synthesis does not alter the chemical properties of CuTCNQ, such as its phase I nature that is formed in pristine DMSO. The study also shows that the solvent employed for the synthesis of CuTCNQ may have large implications on the solvophilic nature of the material fabricated, such that tightly packed uniformly distributed structures of CuTCNQ on a Cu surface tend to be more hydrophobic, whereas larger crystals of CuTCNQ randomly distributed on a Cu foil tend to make the surface extremely hydrophilic and easily wettable. Interestingly, the surface property of Cu-supported CuTCNQ catalyst was found to have a direct effect on the utility of CuTCNQ as a catalyst. The CuTCNQ synthesized using 5 mM TCNQ^0^ with 30% *v/v* water was most hydrophilic and showed superior catalytic activity with 95% completion of the ferricyanide reduction reaction achieved within a few minutes. The bisolvent approach presented in the current work establishes water as a universal solvent to improve the crystallization of CuTCNQ and offers avenues to manipulate the crystallization and dissolution of other metal–organic charge-transfer complexes. We note that although the exact mechanism of the influence of cosolvent on the self-organization of CuTCNQ structures is not clear, this work provides clear evidence that an appropriate choice of cosolvent mixture can significantly influence the kinetics of CuTCNQ crystallization and its co-dissolution. In particular, the current work does not shed light on the potential complexation of solvent molecules either on the surface or within the crystal structure of CuTCNQ—while it is a viable possibility, it is currently difficult to elucidate this aspect due to the inability to form large single crystals of CuTCNQ using the proposed strategy. Future efforts may be directed to study single crystal structures of CuTCNQ synthesized in different solvents to obtain greater insights.

## Figures and Tables

**Table 1:** Catalytic performance of CuTCNQ catalyst grown in water–DMSO bisolvent (30% *v/v* water) under different conditions.

Sample	K_app_ [min^−1^]	Time Taken for 95% Reaction Completion [min]
CuTCNQ 0% H_2_O	0.042	92.5
CuTCNQ 5% H_2_O	0.048	81
CuTCNQ 15% H_2_O	1.03	4
CuTCNQ 30% H_2_O	1.28	2.5

## Data Availability

No new data were created or analyzed in this study. Data sharing is not applicable to this article.

## Supplementary Materials

The following are available online at https://www.mdpi.com/article/10.3390/nano11040954/s1, Figure S1: Chemical structure of TCNQ, Figure S2: Chemical structure of CuTCNQ, Figure S3: Digital images of the solutions during CuTCNQ synthesis in DMSO. (a) 5 mM TCNQ solution before exposure to Cu foil, and (b) represents the same solution after the reaction, Figure S4: Low magnification SEM images of CuTCNQ grown on Cu foil using (a) 1 mM, (b) 2.5 mM and (c) 5 mM TCNQ in DMSO with increasing concentration of water (0, 5, 15, 30%), Figure S5: CuTCNQ crystal width with respect to water concentration, Figure S6: EDX mapping of CuTCNQ grown on Cu foil using (a) 1 mM, (b) 2.5 mM and (c) 5 mM TCNQ in DMSO with increasing concentration of water (0, 5, 15, 30%). Red colour corresponds to carbon while green colour indicates nitrogen, both arising from the TCNQ component of CuTCNQ. EDX maps reveal homogeneous distribution of CuTCNQ structures on Cu foil, particularly when higher concentrations of TCNQ^0^ and water are employed during synthesis, Figure S7: UV-Vis absorbance spectra of TCNQ^0^ obtained from (a) 1 mM (b) 2.5 mM and (c) 5 mM TCNQ^0^ in 0, 5, 15 and 30% v/v H_2_O in water/DMSO bisolvent solution before and after the reaction with Cu foil, Figure S8: Water contact angle images of CuTCNQ on Cu foil with respective SEM images (a) 1 mM, (b) 2.5 mM and (c) 5 mM TCNQ in DMSO with increasing concentration of water (0, 5, 15, 30%). The scale bar is 50 µm, Figure S9: UV-visible absorbance spectra representing the time-dependent catalytic reduction of 1 mM ferricyanide in the presence of excess thiosulfate ions by CuTCNQ fabricated using 5 mM TCNQ^0^ in the presence of (a) 0%, (b) 5%, (c) 15% and (d) 30% v/v H_2_O in a water/DMSO bisolvent system, Figure S10: FTIR spectra of CuTCNQ catalysts prepared using 5 mM TCNQ concentration and different v/v water/DMSO concentrations, as acquired after the catalysis reaction.
