# Erratum: Hussain et al. Increased Crystallization of CuTCNQ in Water/DMSO Bisolvent for Enhanced Redox Catalysis. *Nanomaterials* 2021, *11*, 954

**DOI:** 10.3390/nano11112896

**Published:** 2021-10-29

**Authors:** Zakir Hussain, Ayman Nafady, Samuel R. Anderson, Abdullah M. Al-Enizi, Asma A. Alothman, Rajesh Ramanathan, Vipul Bansal

**Affiliations:** 1 Ian Potter NanoBioSensing Facility, NanoBiotechnology Research Laboratory (NBRL), School of Science, RMIT University, P.O. Box 2476, Melbourne, VIC 3000, Australia; s3472654@student.rmit.edu.au (Z.H.); s3239826@student.rmit.edu.au (S.R.A.); 2 Chemistry Department, College of Science, King Saud University, Riyadh 11451, Saudi Arabia; amenizi@ksu.edu.sa (A.M.A.-E.); aaalothman@ksu.edu.sa (A.A.A.)

## Incorrect Funding Number

There is an error that needs to be corrected in the Funding and Acknowledgments statements of [1].

The updated information is provided below:
