# Supplementary material for: Increased Crystallization of CuTCNQ in Water/DMSO Bisolvent for Enhanced Redox Catalysis
Source: Nanomaterials (Basel). 2021 Apr 8;11(4):954. doi: 10.3390/nano11040954 (PMC8068373; doi:10.3390/nano11040954)
Supplement: Supplementary file 1 [file nanomaterials-11-00954-s001.pdf]

nanomaterials

MDPI

Supplementary Materials

Increased Crystallization of CuTCNQ in Water/DMSO Bisolvent for Enhanced Redox Catalysis

Zakir Hussain [1,†], Ayman Nafady [2,*,†], Samuel R. Anderson [1], Abdullah M. Al-Enizi [2], Asma A. Alothman [2], Rajesh Ramanathan [1,*] and Vipul Bansal [1,*]

[1] Ian Potter NanoBioSensing Facility, NanoBiotechnology Research Laboratory (NBRL), School of Science, RMIT University, GPO Box 2476, Melbourne, VIC 3000, Australia; s3472654@student.rmit.edu.au (Z.H.); s3239826@student.rmit.edu.au (S.R.A.)

[2] Chemistry Department, College of Science, King Saud University, Riyadh 11451, Saudi Arabia; amenizi@ksu.edu.sa (A.M.A.-E.); aaalothman@ksu.edu.sa (A.A.A.)

[*] Correspondence: anafady@ksu.edu.sa (A.N.); rajesh.ramanathan@rmit.edu.au (R.R.); vipul.bansal@rmit.edu.au (V.B.); Tel.: +61-3-9925-2887 (R.R.); +61-3-9925-2121 (V.B.)

[†] These authors contributed equally to this paper.

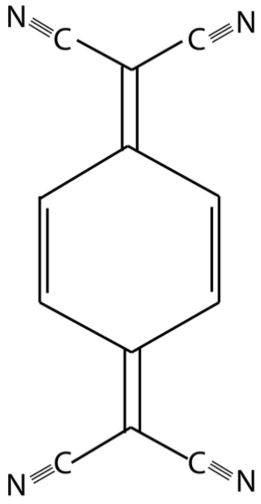

Figure S1. Chemical structure of TCNQ.

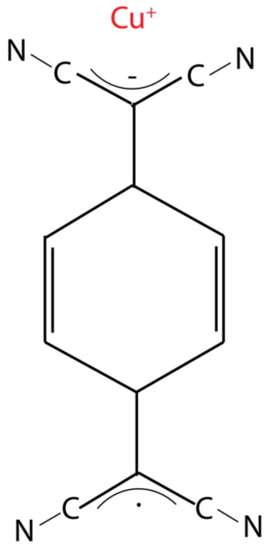

Figure S2. Chemical structure of CuTCNQ.

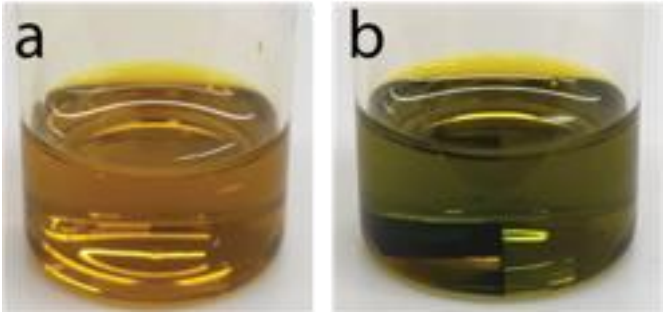

Figure S3. Digital images of the solutions during CuTCNQ synthesis in DMSO. (**a**) 5 mM TCNQ solution before exposure to Cu foil, and (**b**) represents the same solution after the reaction.

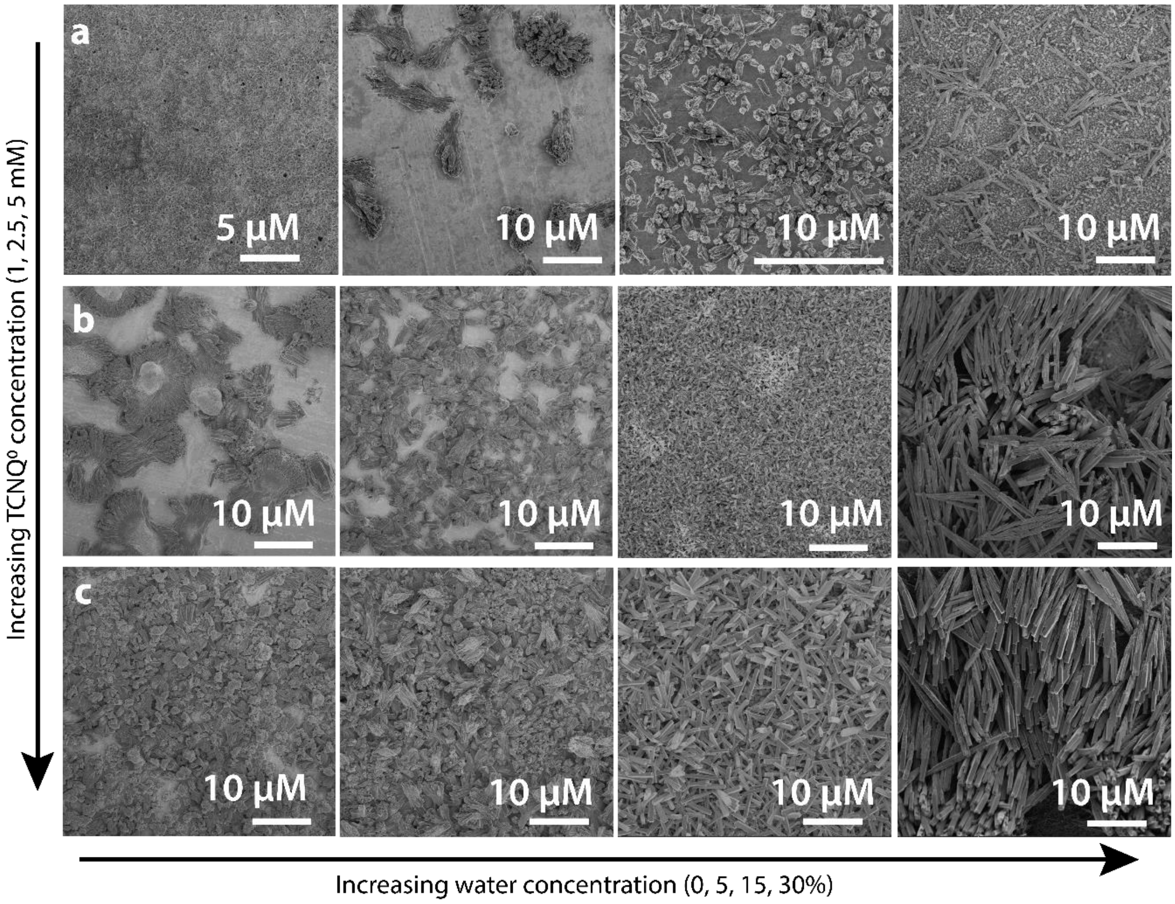

Figure S4. Low magnification SEM images of CuTCNQ grown on Cu foil using (**a**) 1 mM, (**b**) 2.5 mM and (**c**) 5 mM TCNQ in DMSO with increasing concentration of water (0, 5, 15, 30%).

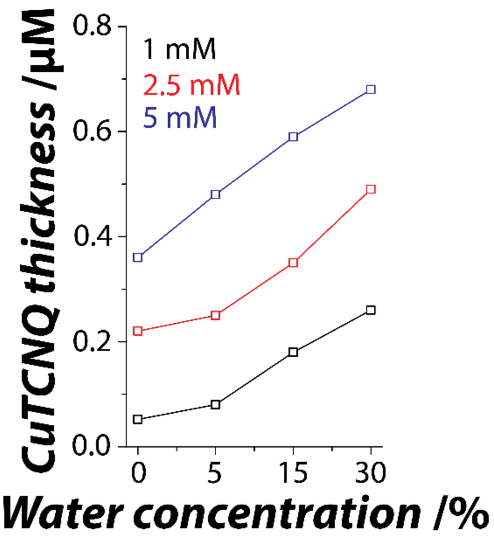

Figure S5. CuTCNQ crystal width with respect to water concentration.

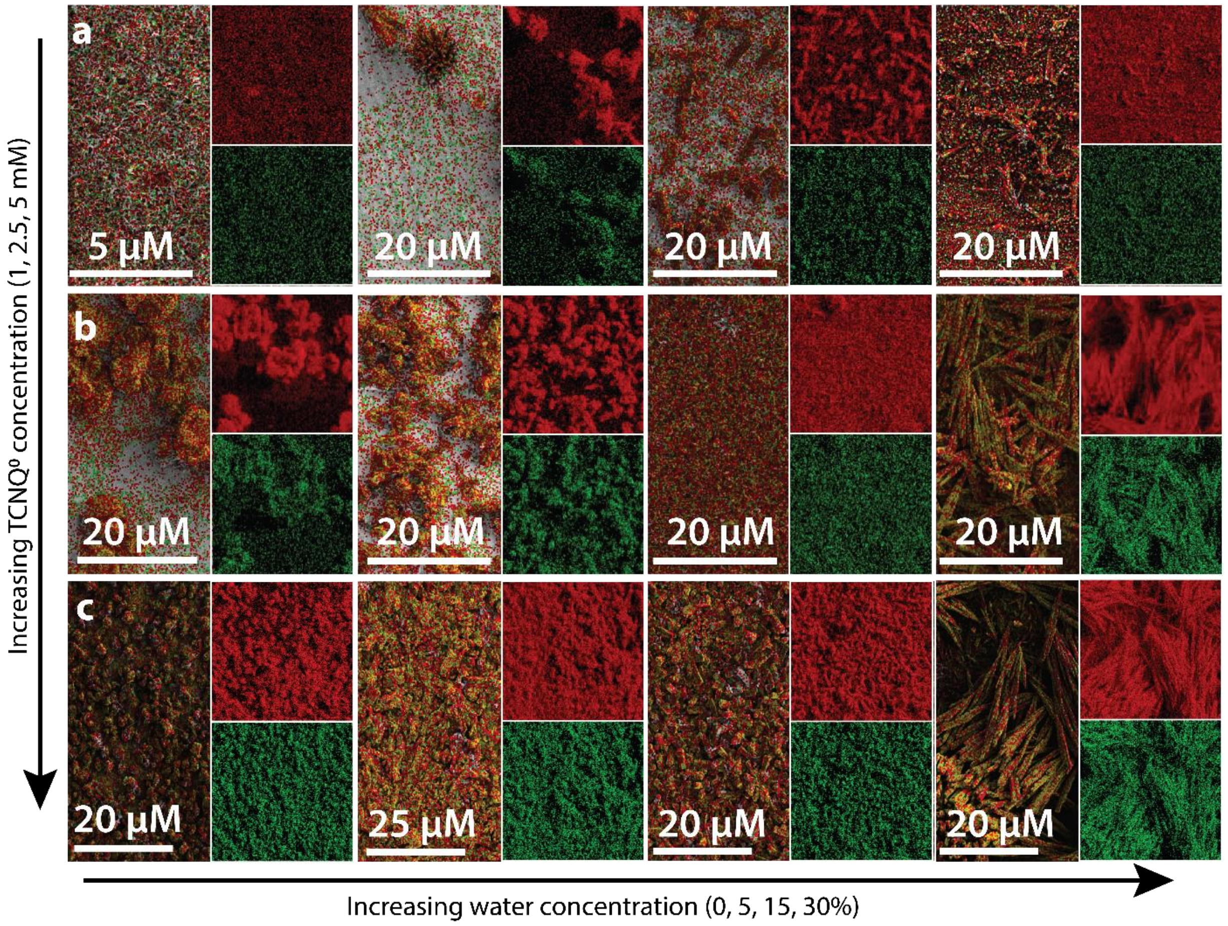

Figure S6. EDX mapping of CuTCNQ grown on Cu foil using (**a**) 1 mM, (**b**) 2.5 mM and (**c**) 5 mM TCNQ in DMSO with increasing concentration of water (0, 5, 15, 30%). Red colour corresponds to carbon while green colour indicates nitrogen, both arising from the TCNQ component of CuTCNQ. EDX maps reveal homogeneous distribution of CuTCNQ structures on Cu foil, particularly when higher concentrations of $TCNQ^0$ and water are employed during synthesis.

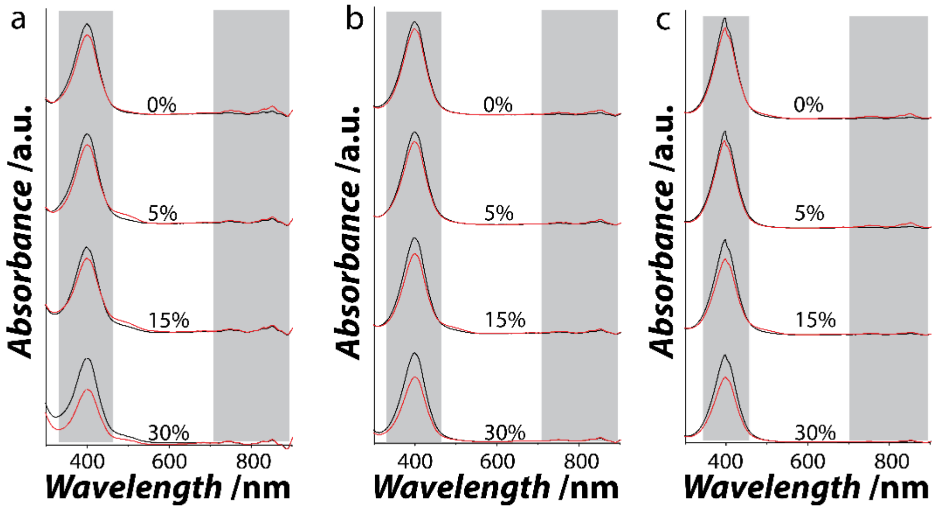

Figure S7. UV-Vis absorbance spectra of $TCNQ^0$ obtained from (**a**) 1 mM (**b**) 2.5 mM and (**c**) 5 mM $TCNQ^0$ in 0, 5, 15 and 30% v/v H_2O in water/DMSO bisolvent solution before and after the reaction with Cu foil.

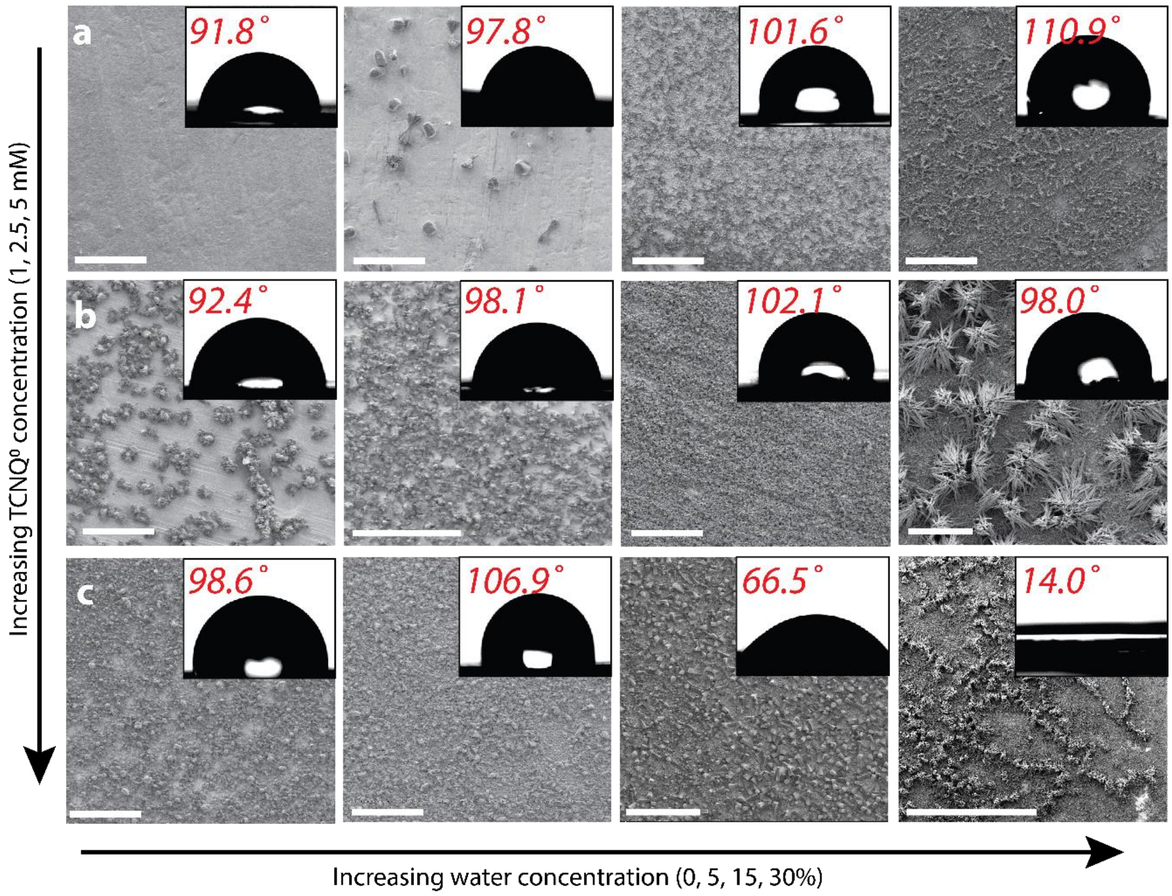

Figure S8. Water contact angle images of CuTCNQ on Cu foil with respective SEM images (**a**) 1 mM, (**b**) 2.5 mM and (**c**) 5 mM TCNQ in DMSO with increasing concentration of water (0, 5, 15, 30%). The scale bar is 50 μm.

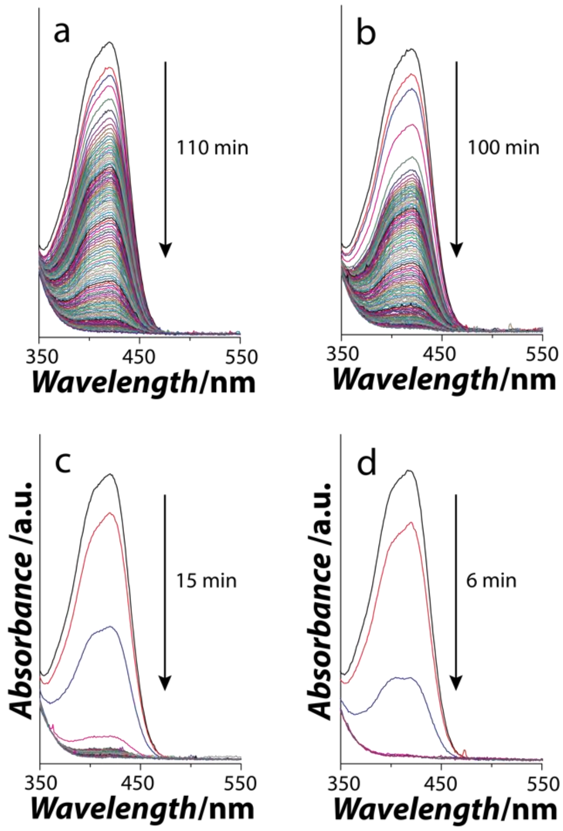

Figure S9. UV-visible absorbance spectra representing the time-dependent catalytic reduction of 1 mM ferricyanide in the presence of excess thiosulfate ions by CuTCNQ fabricated using 5 mM $TCNQ^0$ in the presence of (**a**) 0%, (**b**) 5%, (**c**) 15% and (**d**) 30% v/v H_2O in a water/DMSO bisolvent system.

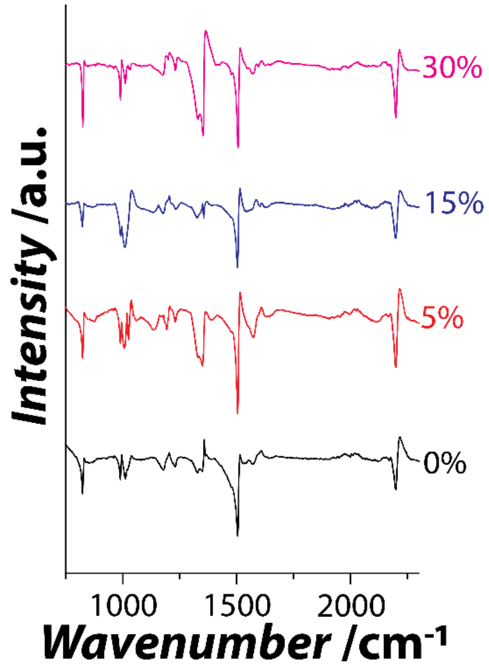

Figure S10. FTIR spectra of CuTCNQ catalysts prepared using 5 mM TCNQ concentration and different v/v water/DMSO concentrations, as acquired after the catalysis reaction.
